# Use of novel lab assays to examine the effect of pyrethroid-treated bed nets on blood-feeding success and longevity of highly insecticide-resistant *Anopheles gambiae* s.l. mosquitoes

**DOI:** 10.1186/s13071-022-05220-y

**Published:** 2022-03-28

**Authors:** Priscille Barreaux, Jacob C. Koella, Raphael N’Guessan, Matthew B. Thomas

**Affiliations:** 1 Liverpool School of Tropical Medicine, Liverpool, UK; 2 Pennsylvania State University, State College, PA USA; 3 University of Neuchâtel, Neuchâtel, Switzerland; 4 London School of Tropical Medicine, London, UK; 5 Vector Control Product Evaluation Centre, Institute Pierre Richet, Bouaké, Côte d’Ivoire; 6 University of York, York, UK

**Keywords:** *Anopheles gambiae*, Pyrethroid resistance, Sublethal effect, Vector control, Blood feeding inhibition, Delayed mortality

## Abstract

**Background:**

There is a pressing need to improve understanding of how insecticide resistance affects the functional performance of insecticide-treated nets (ITNs). Standard WHO insecticide resistance monitoring assays are designed for resistance surveillance and do not necessarily provide insight into how different frequencies, mechanisms or intensities of resistance affect the ability of ITNs to reduce malaria transmission.

**Methods:**

The current study presents some novel laboratory-based assays that attempt to better simulate realistic exposure of mosquitoes to ITNs and to quantify impact of exposure not only on instantaneous mortality, but also on blood-feeding and longevity, two traits that are central to transmission. The assays evaluated the performance of a standard ITN (Permanet® 2.0; Vestergaard Frandsen), a ‘next generation’ combination ITN with a resistance-breaking synergist (Permanet® 3.0) and an untreated net (UTN), against field-derived *Anopheles gambiae* sensu lato mosquitoes from Côte d’Ivoire exhibiting a 1500-fold increase in pyrethroid resistance relative to a standard susceptible strain.

**Results:**

The study revealed that the standard ITN induced negligible instantaneous mortality against the resistant mosquitoes, whereas the resistance-breaking net caused high mortality and a reduction in blood-feeding. However, both ITNs still impacted long-term survival relative to the UTN. The impact on longevity depended on feeding status, with blood-fed mosquitoes living longer than unfed mosquitoes following ITN exposure. Exposure to both ITNs also reduced the blood-feeding success, the time spent on the net and blood-feeding duration, relative to the untreated net.

**Conclusion:**

Although a standard ITN did not have as substantial instantaneous impact as the resistance-breaking net, it still had significant impacts on traits important for transmission. These results highlight the benefit of improved bioefficacy assays that allow for realistic exposure and consider sub- or pre-lethal effects to help assess the functional significance of insecticide resistance.

**Graphical Abstract:**

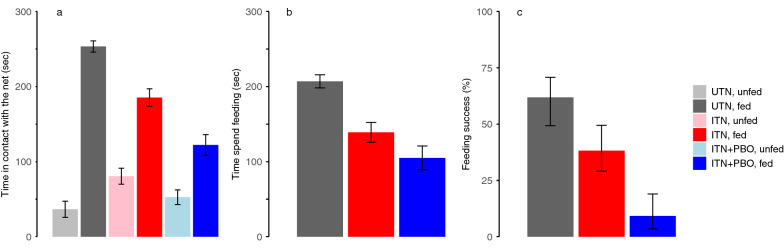

**Supplementary Information:**

The online version contains supplementary material available at 10.1186/s13071-022-05220-y.

## Background

In recent decades, large-scale implementation of pyrethroid-based control tools that target the adult mosquito vectors have helped reduce the burden of malaria [1]. Insecticide-treated nets (ITNs) are the most widely distributed and perhaps most important tool to date [2]. However, their extensive use has led to the evolution of insecticide resistance in many mosquito populations [3–7], and there are now mounting concerns that resistance will render ITNs ineffective and lead to a resurgence of malaria [8–10]. As yet, however, the link between emergence of different resistance mechanisms and intensities of insecticide resistance and control failure remains unclear [11–15], which challenges the development of appropriate resistance mitigation strategies [16].

Mosquito populations are classified as resistant using standardized WHO testing procedures that measure the level of mortality within 24 h of exposure to a diagnostic dose of insecticide [17]. However, this focus on instantaneous mortality ignores possible pre- or sub-lethal effects of ITNs on longevity and blood-feeding success, two important parameters influencing malaria transmission potential [18]. Evidence suggests that ITNs can potentially reduce the transmission of malaria even in the absence of rapid knockdown and death, providing they reduce mosquito longevity and limit the number of mosquitoes that live long enough to enable the malaria parasite to complete its extrinsic incubation period [18–20]. This “sub-lethal” effect of insecticide might be minimal in some conditions [21] or may be enhanced by repeated exposures [18, 22]. In addition, previous studies with susceptible mosquito strains suggest that certain pyrethroid insecticides act on mosquito host-seeking and blood-feeding behavior, by irritating them upon contact or repelling them prior to net contact [23, 24]. While, over short distances, the repellent effect of pyrethroid seems to impact resistant mosquito strains [25–28], it is still unclear whether resistance alters the impact of ITNs on blood-feeding inhibition. In fact, some recent laboratory studies suggest that pyrethroids might even enhance host searching in resistant mosquitoes [29, 30].

**Fig. 1:**
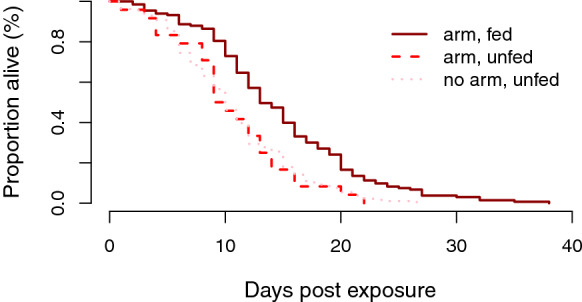
Survival curves for mosquitoes exposed in WHO bioassay tubes against a Permanet® 2.0-treated ITN for 1, 3 or 5 min and with or without access to a human host (arm) in experiment 1. The 3 lines represent the survival curves of mosquitoes that took a blood meal when they had access to a human arm (solid dark-red line), mosquitoes that did not take a blood meal while having access to a human arm (broken red line) or mosquitoes with no access to a blood source (dotted pink line). Experimental blocks 3 and 4 are not represented in this figure as no data are available for exposure times 1 and 3 min

In the study reported here we used two novel assay methods to explore the effects of ITN exposure on initial mortality, blood-feeding inhibition and longevity against field-derived populations of *Anopheles gambiae* sensu lato (s.l.) from central Cote d’Ivoire that are known to exhibit intense resistance to pyrethroids [4]. The primary aim was to examine the sub-/pre-lethal effects of ITNs considering different patterns of exposure. A secondary aim was to explore some novel assays that could possibly be used to supplement the standard WHO assays used to determine insecticide resistance and characterize the bioefficacy of ITNs, ultimately to provide a better assessment of the functional significance of insecticide resistance (Fig. 1).

## Methods

### Mosquito populations

The study used *An. gambiae* s.l. mosquitoes collected in natural breeding habitats around the villages of Yao Koffikro and M’be in central Côte d’Ivoire [31]. In this region, the resistance of mosquito populations to deltamethrin is > 1500-fold higher than that of a standard susceptible strain (Kisumu) [4, 32]. Among other resistance mechanisms, they carry the L1014F knockdown resistance (kdr) (≥ 90% fixed) and N1575Y mutations and upregulate the *CYP6M2*, *CYP6P3* and *CYP9K1* genes [4]. The field-collected larvae were reared at 27 ± 2 °C, 60 ± 20% relative humidty and ambient light, in plastic boxes of 300 larvae with 1 l of deionized water and fed daily with fish food (TetraMin™ Baby; Tetra Werke, Melle, Germany) following a standardized “high food” regime described in [33]. Adult mosquitoes from both villages were combined and kept in 32.5 × 32.5 × 32.5-cm mosquito cages and maintained on a 10% sugar solution.

### Human host preparation

The experimenter (PB) avoided tobacco, alcohol and the use of scented products for 12 h before and during testing. Her arms were washed with unscented soap and rinsed with water the morning before a test. PB’s temperature was monitored to reduce risk of exposing mosquitoes to any active pathogen infection (at no point during the study was PB infected with malaria).

### Bed nets

The experimental treatments consisted of three types of polyester bed nets: (i) unwashed Permanet® 2.0-treated net (ITN; Vestergaard Frandsen, Lausanne, Switzerland); (ii) the roof of Permanet® 3.0-treated net (ITN + PBO); (iii) and an untreated net (UTN) (Coghlan’s, Winnipeg, MB, Canada). The ITN is coated with deltamethrin at a target dose of 55 mg/m^2^ (± 25%). The ITN + PBO is coated with deltamethrin and piperonyl butoxide (PBO) at a target dose of 120 mg/m^2^ (± 25%) and 750 mg/m^2^ (± 25%), respectively [34]. It is worth noting that the difference between the standard ITN and the ITN + PBO product is not just the addition of PBO but also an increased concentration of the active ingredient, deltamethrin for the combination-treated net. Prior to testing, fully susceptible mosquitoes (Kisumu strain) were exposed to samples of netting in WHO tubes (see "General methods" section); all were killed within 24 h when exposed to the treated netting, while none of these exposed to the UTN were killed.

### General methods

The assays used 4- to 5-day-old adult female mosquitoes selected at random from the stock cages. Mosquitoes were assigned haphazardly to a net treatment to provide balanced sample sizes (Additional file 1: Table A). They were starved 4 h prior to testing, with assays conducted in the afternoon during daylight. Following exposure, feeding status was recorded; mosquitoes with a visible amount of bright red blood in their abdomen were considered as “fed”. Following exposure, mosquitoes were kept individually in transparent plastic cups covered with untreated netting, and mortality was recorded daily until all mosquitoes had died. Females had continuous access to a 10% sugar solution and to an egg-laying substrate, although egg numbers were not recorded in order to minimize daily mosquito handling.

### Forced exposure in modified WHO tubes

**Fig. 2:**
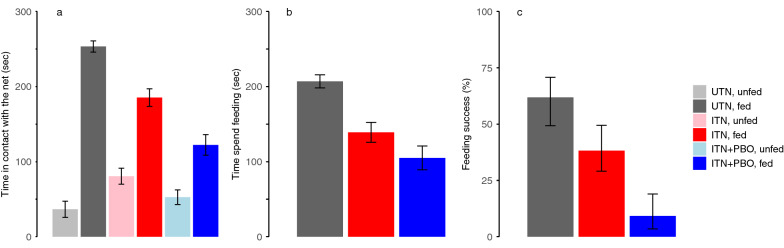
Panel representing the **a** mean time (in seconds) spent on the net, **b** mean time (in seconds) spent taking a blood meal and **c** percentage of feeding success (in percentage) for mosquitoes exposed in a plastic cup to the Permanet® 2.0 (ITN) treatment, the Permanet® 3.0 (ITN + PBO) treatment or treatment with an untreated net (UTN) for 5 min, and with access to a human host in experiment 2. The black and gray bars represent mosquitoes exposed to an UTN that did or did not take a blood meal, respectively. The red and pink bars represent mosquitoes exposed to an ITN that did or did not take a blood meal, respectively. The blue and light-blue bars represent mosquitoes exposed to an ITN + PBO that did or did not take a blood meal, respectively. The replicate 4 is not represented in this figure as no data are available for ITN + PBO. Error bars:** a**,** b** ± standard error;** c** 95% confidence interval

In this assay we examined the effect of a forced exposure to an ITN (PermaNet® 2.0) on mosquito mortality and capacity to blood-feed (Additional file 2: Dataset S1). The aim was partly to determine whether the highly resistant wildtype mosquitoes suffered obvious direct effects from forced contact with the ITN, but also to serve as something of a range finder to determine the effects of duration exposure and the relative influence of blood-feeding on survival following exposure, in order to inform the design of the following experiment. In two experimental blocks (*n*_1_ = 219 and *n*_2_ = 122), mosquitoes were individually exposed to the PermaNet® 2.0 for 1, 3 or 5 min using WHO tubes lined (inner wall and ends) with netting to force the mosquitoes to come into contact with the treated surface. During the exposure, half of the mosquitoes in each block (*n*_1_ = 96 and *n*_2_ = 61) had the opportunity to take a blood meal by feeding on PB’s arm through the netting; the other half were not allowed to feed, but PB held her arm 1 cm away from the tube to provide equivalent host cues. In two additional experimental blocks (*n*_3_ = 84 and *n*_4_ = 56), mosquitoes were exposed for 5 min only (Fig. 2).

### Variable exposure via individual feeding choice

This assay relaxed the forced contact experienced in the WHO tubes by placing mosquitoes individually in transparent 180-ml plastic cups with the top covered with netting, either ITN, ITN + PBO (roof of the Permanet®-3.0 treated netting) or UTN. PB’s arm was placed onto the net at the top of the cup to attract mosquitoes and enable blood-feeding (Additional file 3: Dataset S2). The aim was to simulate more natural patterns and durations of contact during a 5-min exposure period. In principle, if mosquitoes were repelled by the netting or suffered from irritancy following initial contact, they might have only minimal contact. On the other hand, if they were motivated to feed and were unaffected by the presence of the netting, contact could last for up to 5 min. The time mosquitoes were in contact with the net and the duration of their blood meal were recorded, together with subsequent longevity. The ITN, ITN + PBO and a UTN were compared in three experimental blocks (*n*_1_ = 61, *n*_2_ = 58 and *n*_3_ = 127) while in a fourth experimental block (*n*_4_ = 123), females were not tested against the ITN + PBO because of limited mosquito numbers from the larval collection and the desire to increase the sample sizes (Additional file 4: Table C).

### Statistical analysis

All analyses and graphs were done in R version 3.6.1 (available in opensource: https://www.R-project.org/). Contrasts among treatments were assessed with the multcomp package version 1.4–10 and the function ‘glht’ with Tukey’s honestly significant difference test. All complete statistical analyses can be found in Additional file 5: Table B.

#### Forced exposure in modified WHO tube assays

Using a generalized linear model (GLM) with a binomial distribution, the blood-feeding success of mosquitoes given access to a blood source was analyzed to investigate whether the proportion that fed depended on the duration of insecticide exposure and the experimental block.

Survival post exposure was analyzed with a weighted Cox regression (using the R package coxphw due to the violation of the proportional hazards assumption in a Cox regression model) according to the blood-feeding categories (no access to a blood source; access to a blood source but unfed; access to a blood source and fed), the duration of insecticide exposure, the experimental blocks and their interaction. Given the complex interaction found in this analysis and the 5-min time needed for mosquitoes to engorge blood to repletion [35], we analyzed the survival post exposure for blood-fed and unfed mosquitoes separately with a weighted Cox regression including two exposure time categories (1- and 3-min exposure times compared to 5-min exposure time), the experimental blocks and their interaction. In a preliminary model for unfed mosquitoes, whether mosquitoes had access to an arm or not during exposure did not influence longevity; thus, this factor it was not included in the final analysis.

In addition, mosquito survival post exposure in two additional replicates was analyzed together with the other experimental blocks considering mosquitoes exposed in WHO tubes for 5 min only. A weighted Cox model was used to investigate the effect of the blood-feeding categories, the experimental blocks and their interaction.

#### Variable exposure via individual feeding choice

We analyzed the time spent on the net with a Gaussian GLM and an identity link function including the type of bed net (UTN, ITN, ITN + PBO) and the experimental blocks as nominal factors.

We analyzed the time spent feeding with a Gaussian GLM (for fed mosquitoes only) and the proportion of mosquitoes that fed with a binomial GLM, with both analyses including the type of bed nets, the time spent on the net, their interaction and the experimental blocks as nominal factors (the latter in interaction with the other parameters for the binomial GLM).

We analyzed the survival post exposure of the mosquitoes with a weighted Cox proportional hazards model, with the type of bed net, the feeding status, their interactions and the experimental blocks as factors. Considering fed mosquitoes alone, the same analysis was done without the feeding status and adding the time spent on the net and the time spent feeding. We then repeated that analysis for unfed mosquitoes alone with the time spent on the net (summary in Additional file 2: Table B.a.3).

The analysis for the time spent on the net, feeding success and survival post exposure were repeated with an additional experimental block for UTN and ITN treatments only.

## Results

### Forced exposure in modified WHO tube assays

Forced exposure to the ITN resulted in negligible mortality of highly resistant mosquitoes within 24 h regardless of exposure period (note this contrasts to 100% mortality of the susceptible Kisumu strain in pilot studies referred to in " Methods" section) (Fig. 1). In addition, 84.7% (95% confidence interval [CI]: 78.1–89.9%) of mosquitoes that were provided access to a blood meal were able to feed, irrespective of the duration of the exposure (*χ*^2^ = 1.32,* df* = 1, *P* = 0.25). Blood-feeding increased mosquito longevity by approximately 4 days, with blood-fed mosquitoes having a mean (± standard error [SE]) survival time (post exposure at 4 days old) of 14.7 ± 0.59 days post exposure, compared with 10.5 ± 1.04 days post exposure for unfed mosquitoes having access to the arm and 10.7 ± 0.39 days post exposure for unfed mosquitoes having no access to the arm (*χ*^2^ = 29.80,* df* = 1, *P* < 0.001) (Fig. 1). At exposure times of 1 and 3 min, fed mosquitoes lived an average of 2.8 more days post exposure than unfed mosquitoes (unfed: 11.1 ± 0.47 days; fed: 13.9 ± 0.59 days), and when exposed for 5 min, fed mosquitoes lived an average of 6.5 more days post exposure (unfed: 9.8 ± 0.58 days; fed: 16.3 ± 1.36 days) (*χ*^2^ = 14.64,* df* = 1, *P* < 0.001). While the interaction between exposure time and blood-feeding was significant on longevity, there was no significant effect of exposure duration itself (*χ*^2^ = 0.56,* df* = 1, *P* = 0.45). Subgroup analysis showed no influence of exposure duration on mean longevity for unfed mosquitoes (*χ*^2^ = 3.20,* df* = 1, *P* = 0.07). However, longer exposure to insecticide led to a longer life post exposure for blood-fed mosquitoes (13.9 ± 0.59 days for 1- and 3-min exposure and 16.3 ± 1.36 days for 5-min exposure) (*χ*^2^ = 5.78,* df* = 1, *P* = 0.02).

Two additional experimental blocks, in which the exposure time of mosquitoes was 5 min only, corroborated these results. Combining all experimental blocks for the 5-min exposure time showed that a blood meal extended the lifespan of mosquitoes by around 7 days post exposure (9.0 ± 0.73 days for females with no access to blood source; 9.7 ± 0.43 days for those with access to the blood source but unfed; and 16.6 ± 0.87 days for those with access to the blood source and fed) (*χ*^2^ = 51.03,* df* = 1, *P* < 0.001).

## Variable exposure via individual feeding choice

The presence of insecticide reduced the average contact time with the netting (*F* = 21.97,* df* = 2, *P* < 0.001; Fig. 2a). Mosquitoes exposed to the UTN had an average contact time of 167.7 ± 13.06 s, while those exposed to the ITN or ITN + PBO had average contact times of 121.4 ± 9.5 and 59.1 ± 9.25 s, respectively (Tukey pairwise comparisons: *P*_ITN-UTN_ = 0.005, *P*_ITN+PBO-UTN_). The average contact time for the synergist-treated net (ITN + PBO) was lower than that for the ITN without PBO (*P*_ITN+PBO-ITN_ < 0.001).

The feeding duration showed a similar pattern between net types (*F* = 45.30,* df* = 2, *P* < 0.001; Fig. 2b), with average times of 219.3 ± 8.82, 135.4 ± 9.80 and 105.0 ± 15.85 s for UTN, ITN, ITN + PBO, respectively (Tukey pairwise comparisons: *P*_ITN-UTN_ and *P*_ITN+PBO-UTN_ < 0.001, *P*_ITN+PBO-ITN_ = 0.48). Blood-feeding duration was longer with longer net contact time (*F* = 72.21,* df* = 1, *P* < 0.001), independently of the net treatment (*F* = 2.36,* df* = 2, *P* = 0.10).

The presence of insecticide reduced the percentage of mosquitoes that fed successfully. With the UTN, 60.5% (95% CI: 49.3–70.8%) of mosquitoes took a blood meal, while blood-feeding rates were only 38.9% (95% CI: 29.1–49.5%) and 9.2% (95% CI: 3.46–19.0%) with the ITN and ITN + PBO treatments, respectively (*χ*^2^ = 45.70,* df* = 2, *P* < 0.001; Fig. 2c). Blood-fed females spent 3.6-fold more time in contact with nets than unfed ones (unfed: 59.8 ± 6.22 s; fed: 218.7 ± 7.46 s] (χ^2^ = 110.22,* df* = 1, *P* < 0.001). Contact times of the subset of mosquitoes that successfully took a blood meal showed a similar pattern between net types, with average contact times of 253.4 ± 7.49, 185.4 ± 11.73 and 122.3 ± 13.68 s for the UTN, ITN, ITN + PBO treatments, respectively (Tukey pairwise comparisons: *P*_ITN-UTN_ and *P*_ITN+PBO-UTN_ < 0.001, and *P*_ITN+PBO-ITN_ = 0.29). However, unfed mosquitoes exposed to an ITN spent more time in contact with the net compared to those exposed to an UTN, with an average contact time of 36.5 ± 10.82 s for the UTN versus 80.6 ± 10.66 s for the ITN and 52.7 ± 9.73 s for the ITN + PBO (*F* = 11.97,* df* = 2, *P* = 0.002; Tukey pairwise comparisons: *P*_ITN-UTN_ = 0.04 and *P*_ITN+PBO-UTN_ = 0.88, and *P*_ITN+PBO-ITN_ = 0.24). In one experimental block, the feeding success was slightly lower compared to the other blocks (*F* = 5.63,* df* = 1, *P* = 0.02). Thus, while there was no difference in the time spent on the net for blood-fed mosquitoes, there was some variability between two experimental blocks for the contact time of unfed mosquitoes (*F* = 7.92,* df* = 1, *P* = 0.005).

**Fig. 3:**
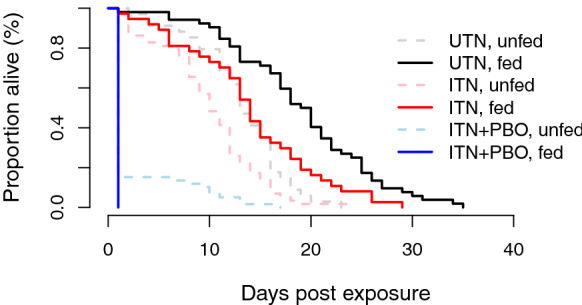
Survival curves for mosquitoes exposed in a plastic cup to Permanet® 2.0-treated netting (ITN), the roof of a Permanet® 3.0-treated netting (ITN + PBO) or an untreated net (UTN) for 5 min, and with access to a human host in experiment 2. The dotted lines and shaded colors represent the survival of mosquitoes that did not take any blood meal even though they had access to a human arm. The solid lines and darker colors represent mosquitoes that successfully fed. The blue lines represent mosquitoes exposed to the ITN + PBO treatment; the red lines, mosquitoes exposed to the ITN treatment; and the black lines, mosquitoes exposed to the UTN treatment. Experimental block 4 is not represented in this figure as no data are available for the ITN + PBO treatment

As observed in the first experiment, there was negligible mortality within 24 h in the UTN and ITN treatments. The ITN + PBO treatment, however, caused a substantial mortality within 24 h of exposure of 86.1% ((95 CI: 75.3–93.5%). Beyond the instantaneous effects, insecticide exposure led to a reduction in long-term survival (*χ*^2^ = 146.87,* df* = 2, *P* < 0.001) (Fig. 3). Mosquitoes exposed to an UTN had an average survival time (post exposure at 4–5 days old) of 16.7 ± 0.74 days; those exposed to the ITN, 11.5 ± 0.62 days; and those exposed to the ITN + PBO, just 2.3 ± 0.44 days. Blood-feeding increased overall longevity (*χ*^2^ = 24.53,* df* = 1, *P* < 0.001) by approximately 6 days for the UTN treatment and 4 days for the ITN treatment. Nonetheless, blood-fed females died more quickly after an exposure to insecticide than those exposed to a UTN (14.0 ± 1.10 days for the ITN vs 19.0 ± 1.00 days for the UTN). For the ITN + PBO treatment, blood-fed mosquitoes had a marginally shorter lifespan than their nonblood-fed counterparts (average survival time post exposure of 1.0 ± 0 days and 2.5 ± 0.49 days, respectively; *χ*^2^ = 10.21,* df* = 2, *P* = 0.006). The time that blood-fed mosquitoes spent on the net and feeding duration did not influence longevity. However, fed mosquitoes exposed to an UTN had a longer lifespan when they spend more time blood-feeding, which was not the case for fed mosquitoes exposed to insecticide (*χ*^2^ = 6.95,* df* = 2, *P* = 0.03).

One additional experimental block was added to an analysis comparing the effects of the ITN against the UTN only (providing 4 blocks in total for this comparison (Additional file 1: Table A)). The presence of insecticide reduced the mean time spent on the net (119.6 ± 7.73 s for the ITN vs 173.3 ± 10.11 s for the UTN) (*F* = 18,02,* df* = 1, *P* < 0.001). Exposure to the ITN led to a significant reduction in blood-feeding (*χ*^2^ = 17.20,* df* = 1, *P* < 0.001), with 38.2% (95% CI 30.6–46.3%) blood-fed mosquitoes in the ITN treatment compared with 61.9% (95% CI: 53.6 to 69.8) in the UTN treatment. Mosquitoes that spent a longer period on the net were proportionally more successful in taking a blood meal (*χ*^2^ = 206.56,* df* = 1, *P* < 0.001). When mosquitoes spent < 1 min on the net, the feeding rate did not differ between the ITN and UTN treatments. However, once contact time exceeded 1 min, blood-feeding increased with contact time for the UTN treatment but did not for the ITN treatment (*χ*^2^ = 11.31,* df* = 1, *P* < 0.001). There was an overall effect of insecticide exposure on longevity (*χ*^2^ = 29.34,* df* = 1, *P* < 0.001). The mean survival time of unfed mosquitoes was 14.0 ± 0.83 days post exposure for the UTN treatment and 10.0 ± 0.50 days post exposure for the ITN treatment, while for fed mosquitoes it was 18.2 ± 0.78 days post exposure and 15.0 ± 0.88 days post exposure, respectively (*χ*^2^ = 42.09,* df* = 1, *P* < 0.001) (Fig. 3).

## Discussion

In the forced exposure assays, a high percentage of mosquitoes were able to blood-feed, and increased contact time with the ITN for up to 5 min was not obviously a factor that would result in death of the mosquitoes compared to a contact time of 1 min. On the contrary, those mosquitoes that did blood-feed lived longer, and a longer contact time with the ITN increased their survival post-exposure. We expected that a longer exposure to insecticide would decrease longevity, but instead we found a clear benefit of having more time to complete a blood meal for survival post-exposure. Other studies have also shown that ITNs fail to cause instantaneous mortality [3, 5] or fully prevent blood-feeding [3, 24, 32] against highly resistant mosquitoes, suggesting a loss of personal protection due to resistance. However, the feeding choice exposure assays provide a slightly more nuanced picture and showed that the ITN did reduce the proportion of mosquitoes that fed successfully. Unfed mosquitoes were found to spend more time in contact with the ITN than their unfed counterparts exposed to the UTN, yet fewer mosquitoes exposed to the ITN ultimately fed. This result suggests that reduced feeding was not due to the mosquitoes avoiding the net or being repelled by it, but more likely because contact with the insecticide reduced feeding capacity. In turn, insecticide exposure reduced the time spent on the net feeding. Spending less time on a net during blood-feeding does not necessarily mean that the size of the blood meal is smaller and/or insufficient for malaria transmission [36]. The presence of insecticide might reduce the capacity to engorge blood [24], or it could be hypothesized that mosquitoes take the same amount of blood in a shorter period in order to minimize their contact time with the treated net. A shorter time in contact with the treated net could lower the insecticide dosage received by blood-feeders, and this could be a behavioral adaptation of mosquitoes living in areas with a high use of ITNs. Whether similar results are observable in mosquitoes infected with malaria parasites is unclear. It is known that malaria infection alters feeding rates and blood-seeking behaviors [37, 38], but more research is needed to understand whether insecticide exposure impacts vector competence [39–42] and/or whether the presence of malaria parasites affects the expression of insecticide resistance [29, 43].

Regardless of the exposure pattern/duration there was negligible mosquito mortality within 24 h of contact with an ITN. However, this standard 24-h assessment [17] misses potential long-term effects of exposure. Data from both assays show reduced long-term survival following exposure to a standard ITN, a result consistent with the delayed mortality for highly insecticide-resistant mosquitoes reported elsewhere [18]. The experiments also highlight that the standard WHO test procedures for evaluating resistance and measuring the bio-efficacy of ITNs [17, 44] are weak indicators of how ITNs ultimately determine malaria transmission risk and, hence, of understanding the functional significance of insecticide resistance. The WHO resistance assay uses tubes (as used in the initial assays here) to force mosquitoes into contact with filter paper treated with diagnostic doses of insecticide for 1 h [45–47], and the WHO ITN bioefficacy assay uses cones to force mosquitoes into contact with ITNs for 3 min [48–50]. Neither assay simulates how mosquitoes contact ITNs during host-searching and blood-feeding in nature [27]. According to data acquired by Diop et al. [51], mosquitoes exposed to insecticide tend to bounce on the ITN until they decide to probe and take a blood meal. How long they choose to stay on the net depends on the level of toxicity of the net as well as on the presence of a host [52]. The cup assay used in the present study provides a potential method to allow for more realistic patterns of contact with an ITN. The data reveal that the repellent and irritant effects of deltamethrin [53, 54], especially in combination with the synergist PBO [55], reduce the time spent on the treated net but do not completely prevent mosquitoes from biting through it. Interestingly, limited repellency may help maximize the sublethal toxic effects of insecticide against them, which would increase ITN efficacy [18, 20].

The data also highlight the importance of blood-feeding in the evaluation of insecticide resistance. In general, those mosquitoes that blood-fed survived insecticide exposure better than those that did not (the exception being in the ITN + PBO exposure). Whether this is because those mosquitoes able to feed during contact with an insecticide are the most resistant and robust individuals, or whether blood-feeding itself enhances expression of insecticide resistance is unclear. The observation that the longevity of mosquitoes having no access to a blood source was similar to that of mosquitoes failing to blood-feed despite the access to a blood source suggests the effect is more to do with blood-feeding than individual variation (i.e. it would be expected that the overall survival of mosquitoes with no access to blood should be greater as these mosquitoes represent a mixed population that includes the potentially more robust individuals). In addition, ingestion of a blood meal induces oxidative stress, leading to increased metabolic activity [56, 57], which could in turn result in the higher expression of detoxification enzymes [58] and perhaps also influence the toxic dose received by mosquitoes exposed to insecticide.

The PBO-treated net was found to have a clear advantage in comparison to the standard ITN, yielding reduced blood-feeding success and increased mortality rate, yet it is unclear whether these effects are due to differences in the concentrations of the active ingredient (the net with PBO has a higher deltamethrin concentration than the standard ITN) or the action of the synergist (PBO) alone. Whatever the mechanism, the results provide an interesting perspective on the significance of resistance relative to different comparators; i.e. the impact depends on whether you consider resistance to be the difference between an ITN and an untreated net (which is like asking how bad an ITN has become), or between an ITN and a resistance-breaking net (which is like asking how much better could an ITN be).

We acknowledge that the present study used only one human host and that attraction and blood meal quality can vary between hosts. It would be interesting for future work to explore the extent to which different hosts lead to variation in patterns of feeding and subsequent lethal and sub-lethal effects of exposure (our expectation is that the qualitative differences between human hosts will likely yield less variation than the difference between the presence or absence of a host). It would also be interesting to further examine possible effects of *Plasmodium* infection on feeding behavior and exposure rates since it is the infected mosquitoes that we ultimately care about.

## Conclusion

Overall, the combination of delayed mortality and antifeedant effects suggests that ITNs retain at least some functionality above and beyond a simple physical barrier, even against mosquitoes with 1500-fold higher resistance. These effects do not mean that ITNs are as effective against resistant mosquitoes as they are against susceptible ones. Moreover, the Permanet® 3.0 (ITN + PBO) treatment induced much greater mortality and feeding inhibition than did the standard ITN treatment, suggesting improved control potential of ‘resistance-breaking’ nets in areas of high insecticide resistance. Nonetheless, the results provide further evidence to support why ITNs might continue to contribute to reduced malaria transmission in the face of insecticide resistance. This is likely to be especially relevant in areas with high effective coverage (i.e. high ownership and use) of ITNs, as even small effect sizes at the individual level can lead to large overall effect sizes when multiplied up to community level [9, 32]. As such, the current study supports the need for further research to fully understand the epidemiological significance of resistance.

## Supplementary Information

**Additional file 1: Table A.** Sample size used for each experimental blocks. Experiment a: forced exposure in modified WHO tubes; Experiment b: variable exposure via individual feeding choice.

**Additional file 2: Dataset 1.** Forced exposure in modified WHO tube assay.

**Additional file 3: Dataset 2.** Variable exposure via individual feeding choice.

**Additional file 4: Table C.** Scripts of the statistical analysis in Rstudio.

**Additional file 5: Table B.** Statistical analysis summaries.

## Data Availability

All datasets generated, used and analyzed in this study are included in this published article.

## Abbreviations

CI: Confidence Interval
GLM: Generalized linear model
ITN: Insecticide-treated net (Permanet® 2.0)
ITN + PBO: Insecticide-treated net with PBO (Permanet® 3.0 roof section)
PB: Priscille Barreaux
PBO: Piperonyl butoxide
UTN: Untreated net
