# Supplementary material for: Use of novel lab assays to examine the effect of pyrethroid-treated bed nets on blood-feeding success and longevity of highly insecticide-resistant Anopheles gambiae s.l. mosquitoes
Source: Parasit Vectors. 2022 Mar 28;15:111. doi: 10.1186/s13071-022-05220-y (PMC8962112; doi:10.1186/s13071-022-05220-y)
Supplement: Supplementary file 1 — Additional file 1: Table A. Sample size used for each experimental blocks. Experiment a: forced exposure in modified WHO tubes; Experiment b: variable exposure via individual feeding choice. [file 13071_2022_5220_MOESM1_ESM.docx]

**Supplementary Information**

**Additional file 1: Table A: Sample size used for each experimental blocks in experiment a.: Forced exposure in modified WHO tubes and b.: Variable exposure via individual feeding choice.** This table also informs about the access to blood, insecticide exposure treatments and exposure time for each treatment given to the tested mosquitoes. ITN: PermaNet® 2.0; ITN+PBO the roof of a PermaNet® 3.0; UTN: untreated net.

| Experiment | Experimental blocks | Access to blood | Bednet | Exposure  time (min) | Sample size | Sample size per experimental blocks (n) |
| --- | --- | --- | --- | --- | --- | --- |
| a. | 1 | Yes  Yes  Yes  No  No  No | ITN | 1 | 34 | 219 |
|  |  |  | ITN | 3 | 32 |  |
|  |  |  | ITN | 5 | 30 |  |
|  |  |  | ITN | 1 | 40 |  |
|  |  |  | ITN | 3 | 40 |  |
|  |  |  | ITN | 5 | 43 |  |
|  | 2 | Yes  Yes  Yes | ITN | 1 | 24 | 122 |
|  |  |  | ITN | 3 | 21 |  |
|  |  |  | ITN | 5 | 16 |  |
|  |  | No  No  No | ITN | 1 | 16 |  |
|  |  |  | ITN | 3 | 21 |  |
|  |  |  | ITN | 5 | 24 |  |
|  | 3 | Yes | ITN | 5 | 46 | 84 |
|  |  | No | ITN | 5 | 38 |  |
|  | 4 | Yes | ITN | 5 | 24 | 56 |
|  |  | No | ITN | 5 | 32 |  |
| b. | 1 | Yes | UTN | 5 | 21 | 61 |
|  |  | Yes | ITN | 5 | 20 |  |
|  |  | Yes | ITN+PBO | 5 | 20 |  |
|  | 2 | Yes | UTN | 5 | 18 | 58 |
|  |  | Yes | ITN | 5 | 20 |  |
|  |  | Yes | ITN+PBO | 5 | 20 |  |
|  | 3 | Yes | UTN | 5 | 47 | 127 |
|  |  | Yes | ITN | 5 | 55 |  |
|  |  | Yes | ITN+PBO | 5 | 25 |  |
|  | 4 | Yes | UTN | 5 | 61 | 123 |
|  |  | Yes | ITN | 5 | 62 |  |

|  |  |  |  |  |
| --- | --- | --- | --- | --- |
