# Supplementary material for: Use of novel lab assays to examine the effect of pyrethroid-treated bed nets on blood-feeding success and longevity of highly insecticide-resistant Anopheles gambiae s.l. mosquitoes
Source: Parasit Vectors. 2022 Mar 28;15:111. doi: 10.1186/s13071-022-05220-y (PMC8962112; doi:10.1186/s13071-022-05220-y)
Supplement: Supplementary file 2 — Additional file 2: Dataset 1. Forced exposure in modified WHO tube assay. [file 13071_2022_5220_MOESM2_ESM.docx]

**Additional file 2: B. Statistical analysis summaries.**

1. **Statistical analysis summaries**

| 1. **Forced exposure in modified WHO tube assays** |  |  |  |  |
| --- | --- | --- | --- | --- |
|  |  |  |  |  |
| **1. Mosquito blood feeding success** |  |  |  |  |
| glm(feeding success~exposure times+experimental blocks, family="binomial") | | |  |  |
|  |  |  |  |  |
| *Fixed effects* | *Df* | *Chisq* | *Pr (>Chi)* |  |
| Exposures times | 1 | 1.32 | 0.25 |  |
| Experimental blocks | 1 | 0.04 | 0.83 |  |
|  |  |  |  |  |
| **2. Mosquito longevity** |  |  |  |  |
| coxph(Surv(days post exposure, death)~blood feeding categories*exposure times*experimental blocks) | | | |  |
|  |  |  |  |  |
| *Fixed effects* | *Df* | *Chisq* | *Pr (>Chi)* |  |
| Blood feeding categories | 1 | 29.80 | < 0.001 |  |
| Exposure times | 1 | 0.56 | 0.45 |  |
| Experimental blocks | 1 | 45.84 | < 0.001 |  |
| Blood feeding categories:exposure times | 1 | 14.64 | <0.001 |  |
| Blood feeding categories:experimental blocks | 1 | 1.04 | 0.31 |  |
| Exposure times:experimental blocks | 1 | 3.45 | 0.06 |  |
| Blood feeding categories:exposure times:experimental blocks | 1 | 0.00 | 0.94 |  |
|  |  |  |  |  |
| **3. Mosquito longevity for unfed mosquitoes** |  |  |  |  |
| coxph(Surv(days post exposure, death)~access to arm*exposure time categories*experimental blocks) | |  |  |  |
|  |  |  |  |  |
| *Fixed effects* | *Df* | *Chisq* | *Pr (>Chi)* |  |
| Exposure time categories | 1 | 3.2 | 0.07 |  |
| Access to arm | 1 | 0.16 | 0.68 |  |
| Experimental blocks | 1 | 41.42 | < 0.001 |  |
| Exposure times:access to arm | 1 | 0.03 | 0.86 |  |
| Exposure times:experimental blocks | 1 | 2.23 | 0.13 |  |
| Access to arm:experimental blocks | 1 | 1.51 | 0.22 |  |
| Exposure times:access to arm:experimental blocks | 1 | 1.73 | 0.19 |  |
|  |  |  |  |  |
| **3. Mosquito longevity for fed mosquitoes** |  |  |  |  |
| coxph(Surv(days post exposure, death)~exposure time categories*experimental blocks |  |  |  |  |
|  |  |  |  |  |
| *Fixed effects* | *Df* | *Chisq* | *Pr (>Chi)* |  |
| Exposure time categories | 1 | 5.78 | 0.02 |  |
| Experimental blocks | 1 | 15.66 | < 0.001 |  |
| Exposure time categories:experimental blocks | 1 | 1.92 | 0.17 |  |
|  |  |  |  |  |
| **4. Mosquito longevity for 5 min exposure only** |  |  |  |  |
| coxph(Surv(days post exposure, censor)~blood feeding categories+experimental blocks | |  |  |  |
|  |  |  |  |  |
| *Fixed effects* | *Df* | *Chisq* | *Pr (>Chi)* |  |
| Blood feeding categories | 1 | 51.03 | < 0.001 |  |
| Experimental blocks | 1 | 0.27 | 0.60 |  |
| Blood feeding categories:experimental blocks | 1 | 0.96 | 0.33 |  |
|  |  |  |  |  |
|  |  |  |  |  |

| 1. **Variable exposure via individual feeding choice** |  |  |  |  |
| --- | --- | --- | --- | --- |
|  |  |  |  |  |
|  |  |  |  |  |
| **1. Time spent on the net** |  |  |  |  |
| glm(time spent of the net~net treatments+experimental blocks, family="gaussian") | | | |  |
|  |  |  |  |  |
| *Fixed effects* | *Df* | *F* | *Pr (>F)* |  |
| Net types | 2 | 21.97 | < 0.001 |  |
| Experimental blocks | 1 | 0.77 | 0.38 |  |
|  |  |  |  |  |
| **2. Blood feeding duration** |  |  |  |  |
| glm(feeding duration~net types*time spent on the net+experimental blocks, family="gaussian") | | | | |
|  |  |  |  |  |
| *Fixed effects* | *Df* | *F* | *Pr (>F)* |  |
| Net types | 2 | 45.30 | < 0.001 |  |
| Time spent on the net | 1 | 72.21 | < 0.001 |  |
| Experimental blocks | 1 | 0.32 | 0.57 |  |
| Net types:time spent on the net | 2 | 2.36 | 0.10 |  |
|  |  |  |  |  |
| **3. Blood feeding success** |  |  |  |  |
| glm(fed~net types*time spent on the net*experimental blocks, family="binomial") | | | |  |
|  |  |  |  |  |
| *Fixed effects* | *Df* | *Chisq* | *Pr (>Chi)* |  |
| Net types | 2 | 45.70 | < 0.001 |  |
| Time spent on the net | 1 | 110.22 | < 0.001 |  |
| Experimental blocks | 1 | 5.63 | 0.02 |  |
| Net types:time spent on the net | 2 | 11.97 | 0.002 |  |
| Net types:experimental blocks | 2 | 1.35 | 0.51 |  |
| Time spent on the net:experimental blocks | 1 | 7.92 | 0.005 |  |
| Net types:time spent on the net:experimental blocks | 2 | 1.19 | 0.55 |  |
|  |  |  |  |  |
| **4. Longevity** |  |  |  |  |
| coxph(Surv(days post exposure, death)~net types*feeding status+experimental blocks) | | | |  |
|  |  |  |  |  |
| *Fixed effects* | *Df* | *Chisq* | *Pr (>Chi)* |  |
| Net types | 2 | 146.87 | < 0.001 |  |
| Feeding status | 1 | 24.54 | < 0.001 |  |
| Experimental blocks | 1 | 3.25 | 0.07 |  |
| Net types:feeding status | 2 | 10.22 | 0.006 |  |
|  |  |  |  |  |
| **5. Longevity for bloodfed mosquitoes alone** |  |  |  |  |
| coxph(Surv(days post exposure, death)~net types*time spent on the net*feeding duration+experimental blocks) | | | | |
|  |  |  |  |  |
| *Fixed effects* | *Df* | *Chisq* | *Pr (>Chi)* |  |
| Net types | 2 | 41.20 | < 0.001 |  |
| Time spent on the net | 1 | 0.05 | 0.81 |  |
| Feeding duration | 1 | 3.63 | 0.06 |  |
| Experimental blocks | 1 | 1.22 | 0.27 |  |
| Net types:time spent on the net | 2 | 2.38 | 0.30 |  |
| Net types:feeding duration | 2 | 6.95 | 0.03 |  |
| Time spent on the net:feeding duration | 1 | 0.25 | 0.62 |  |
| Net types:time spent on the net:feeding duration | 2 | 1.09 | 0.58 |  |
|  |  |  |  |  |
| **6. Longevity for unfed mosquitoes alone** |  |  |  |  |
| coxph(Surv(days post exposure, death)~net types*time spent on the net+experimental blocks | | | | |
|  |  |  |  |  |
| *Fixed effects* | *Df* | *Chisq* | *Pr (>Chi)* |  |
| Net types | 2 | 71.12 | < 0.001 |  |
| Time spent on the net | 1 | 0.02 | 0.89 |  |
| Experimental blocks | 1 | 1.72 | 0.19 |  |
| Net types:time spent on the net | 2 | 1.29 | 0.52 |  |
|  |  |  |  |  |
| **7. Time spent on the net (ITN and UTN treatments alone)** |  |  |  |  |
| glm(time spent on the net~net types+experimental blocks, family="gaussian") | | | |  |
|  |  |  |  |  |
| *Fixed effects* | *Df* | *F* | *Pr (>F)* |  |
| Net types | 1 | 18.02 | < 0.001 |  |
| Experimental blocks | 1 | 0.03 | 0.86 |  |
|  |  |  |  |  |
| **8. Mosquito feeding success (ITN and UTN treatments alone)** | |  |  |  |
| glm(feeding success~net types*time spent on the net+experimental blocks, family="binomial" | | | | |
|  |  |  |  |  |
| *Fixed effects* | *Df* | *Chisq* | *Pr (>Chi)* |  |
| Net types | 1 | 17.20 | < 0.001 |  |
| Time spent on the net | 1 | 206.56 | < 0.001 |  |
| Experimental blocks | 1 | 1.49 | 0.22 |  |
| Net types:time spent on the net | 1 | 11.31 | < 0.001 |  |
|  |  |  |  |  |
| **9. Mosquito longevity (ITN and UTN treatments alone)** |  |  |  |  |
| coxph(Surv(days post exposure, death)~net types*feeding status+experimental blocks) | | | |  |
|  |  |  |  |  |
| *Fixed effects* | *Df* | *Chisq* | *Pr (>Chi)* |  |
| Net types | 1 | 29.34 | < 0.001 |  |
| Feeding status | 1 | 42.09 | < 0.001 |  |
| Experimental blocks | 1 | 0.18 | 0.67 |  |
| Net types:feeding status | 1 | 2.68 | 0.10 |  |
