# Supplementary material for: Use of novel lab assays to examine the effect of pyrethroid-treated bed nets on blood-feeding success and longevity of highly insecticide-resistant Anopheles gambiae s.l. mosquitoes
Source: Parasit Vectors. 2022 Mar 28;15:111. doi: 10.1186/s13071-022-05220-y (PMC8962112; doi:10.1186/s13071-022-05220-y)
Supplement: Supplementary file 3 — Additional file 3: Dataset 2. Variable exposure via individual feeding choice. [file 13071_2022_5220_MOESM3_ESM.docx]

**Supplementary Information**

**Additional file 3: C. Scripts of the statistical analysis in R**^©^**.**

1. **Scripts of the statistical analysis in R**^©^**.**

**###########################################################################**

**Use of novel lab-assays to examine the effect of pyrethroid-treated bed nets on blood**

**feeding success and longevity of highly resistant Anopheles gambiae s.l. mosquitoes.**

**###########################################################################**

####

#setwd("")

#### Statistical analysis ###

## Dataset S1. Forced exposure in modified WHO tube assays

data.S1 <-read.csv("Dataset S1. Forced exposure in modified WHO tube assays.csv",header=TRUE)

attach(data.S1)

summary(data.S1)

## Experimental blocks 1+2

data.S1.subset <- subset(data.S1, subset =="1")

summary(data.S1.subset)

#### 1. Feeding success for those that have access to the arm

new.data.S1.subset <- subset(data.S1.subset, access.to.arm =="arm")

new.data.S1.subset

feeding.success.glm <- glm(feeding.status~exposure.times+experimental.blocks, family="binomial", data=new.data.S1.subset)

summary(feeding.success.glm)

feeding.success.glm

anova(feeding.success.glm,test="Chi")

#### 2. Longevity

library(survival)

library(splines)

modelfix.1<-coxph(Surv(days.post.exposure,death)~blood.feeding.categories*exposure.times*experimental.blocks, data=data.S1.subset)

summary(modelfix.1)

anova(modelfix.1)

#### 3. Longevity for un-fed mosquitoes

new.data2 <- subset(data.S1.subset, feeding.status =="0")

modelfix.2<-coxph(Surv(days.post.exposure,death)~exposure.time.categories*access.to.arm*experimental.blocks, data=new.data2)

summary(modelfix.2)

anova(modelfix.2)

#### 4. Longevity for fed mosquitoes

new.data3 <- subset(data.S1.subset, feeding.status =="1")

modelfix.3<-coxph(Surv(days.post.exposure,death)~exposure.time.categories*experimental.blocks, data=new.data3)

summary(modelfix.3)

anova(modelfix.3)

###########################################################################

## Experimental blocks 1+2+3+4, 5 min only

data.S1.subset.2 <- subset(data.S1, exposure.time.categories =="2")

summary(data.S1.subset.2)

data.S1.subset.2

modelfix.1<-coxph(Surv(days.post.exposure,death)~blood.feeding.categories*experimental.blocks, data=data.S1.subset.2)

summary(modelfix.1)

anova(modelfix.1)

###########################################################################

## Dataset S2. Variable exposure via individual feeding choice

data.S2 <-read.csv("Dataset S2. Variable exposure via individual feeding choice.csv",header=TRUE)

attach(data.S2)

summary(data.S2)

new.data.S2 <- subset(data.S2, subset =="1")

summary(new.data.S2)

#### 1. Time spent on the net

time.net.glm <- glm(time.spent.on.the.net~net.types+experimental.blocks, family="gaussian", data=new.data.S2)

summary(time.net.glm)

time.net.glm

anova(time.net.glm,test="F")

new.data.S2$sxt<- interaction(new.data.S2$net.types)

glm.posthoc <- glm(time.spent.on.the.net~-1 + sxt, data=new.data.S2,"gaussian", weights=rep(10, nrow(new.data.S2)))

time.net <-glht(glm.posthoc,mcp(sxt='Tukey'))

summary(time.net)

cld2 <- cld(glht(glm.posthoc,mcp(sxt = "Tukey")))

cld2

#### 2. Blood feeding duration

new.data4 <- subset(new.data.S2, feeding.status =="1")

summary(new.data4)

time.feed.glm <- glm(feeding.duration~net.types*time.spent.on.the.net+experimental.blocks, family="gaussian", data=new.data4)

summary(time.feed.glm)

time.feed.glm

anova(time.feed.glm,test="F")

new.data4$sxt<- interaction(new.data4$net.types)

glm.posthoc <- glm(feeding.duration~-1 + sxt, data=new.data4,"gaussian", weights=rep(10, nrow(new.data4)))

time.feed <-glht(glm.posthoc,mcp(sxt='Tukey'))

summary(time.feed)

cld2 <- cld(glht(glm.posthoc,mcp(sxt = "Tukey")))

cld2

#### 3. Blood feeding success

feeding.success.glm <- glm(feeding.status~net.types*time.spent.on.the.net*experimental.blocks, family="binomial", data=new.data.S2)

summary(feeding.success.glm)

feeding.success.glm

anova(feeding.success.glm,test="Chi")

new.data.S2$sxt<- interaction(new.data.S2$net.types,new.data.S2$feeding.status)

glm.posthoc <- glm(time.spent.on.the.net~-1 + sxt, data=new.data.S2,"gaussian", weights=rep(10, nrow(new.data.S2)))

time.net <-glht(glm.posthoc,mcp(sxt='Tukey'))

summary(time.net)

cld2 <- cld(glht(glm.posthoc,mcp(sxt = "Tukey")))

cld2

data3$sxt<- interaction(data3$experimental.blocks,data3$fed)

glm.posthoc <- glm(time.spent.on.the.net~-1 + sxt, data=data3,"gaussian", weights=rep(10, nrow(data3)))

time.net <-glht(glm.posthoc,mcp(sxt='Tukey'))

summary(time.net)

cld2 <- cld(glht(glm.posthoc,mcp(sxt = "Tukey")))

cld2

#### 4. Longevity

summary(new.data.S2)

modelfix.4<-coxph(Surv(days.post.exposure,censor)~net.types*feeding.status+experimental.blocks, data=new.data.S2)

summary(modelfix.4)

anova(modelfix.4)

# fed alone

modelfix.5<-coxph(Surv(days.post.exposure,censor)~net.types*time.spent.on.the.net*feeding.duration+experimental.blocks, data=new.data4)

summary(modelfix.5)

anova(modelfix.5)

# unfed alone

new.data5 <- subset(new.data.S2, feeding.status =="0")

modelfix.6<-coxph(Surv(days.post.exposure,censor)~net.types*time.spent.on.the.net+experimental.blocks, data=new.data5)

summary(modelfix.6)

anova(modelfix.6)

##### 4 Replicates comparing ITN and UTN only

data.S2.complete <- subset(data.S2, subset.1 =="1")

summary(data.S2.complete)

#### 1b. Time spent on the net with 4 replicates

time.net.glm <- glm(time.spent.on.the.net~net.types+experimental.blocks, family="gaussian", data=data.S2.complete)

summary(time.net.glm)

time.net.glm

anova(time.net.glm,test="F")

#### 3b. Blood feeding success with 4 replicates

feeding.success.glm <- glm(feeding.status~net.types*time.spent.on.the.net+experimental.blocks, family="binomial", data=data.S2.complete)

summary(feeding.success.glm)

feeding.success.glm

anova(feeding.success.glm,test="Chi")

#### 4b. Longevity with 4 replicates

modelfix.6<-coxph(Surv(days.post.exposure,censor)~net.types*feeding.status+experimental.blocks, data=data.S2.complete)

summary(modelfix.6)

anova(modelfix.6)
